# Prevalence of Multidrug-Resistant Diarrheagenic *Escherichia coli* in Asia: A Systematic Review and Meta-Analysis

**DOI:** 10.3390/antibiotics11101333

**Published:** 2022-09-29

**Authors:** Mohd Zulkifli Salleh, Nik Mohd Noor Nik Zuraina, Khalid Hajissa, Mohamad Ikram Ilias, Zakuan Zainy Deris

**Affiliations:** 1 Department of Medical Microbiology & Parasitology, School of Medical Sciences, Universiti Sains Malaysia Health Campus, Kubang Kerian 16150, Malaysia; 2 Department of Zoology, Faculty of Science and Technology, Omdurman Islamic University, P.O. Box 382, Omdurman 14415, Sudan; 3 Department of Pediatrics, School of Medical Sciences, Universiti Sains Malaysia Health Campus, Kubang Kerian 16150, Malaysia

**Keywords:** *Escherichia coli*, diarrheagenic, antibiotic resistance, epidemiology, Asia, systematic review, meta-analysis

## Abstract

Diarrhea is one of the leading causes of morbidity and mortality in developing countries. Diarrheagenic *Escherichia coli* (DEC) is an important bacterial agent for diarrhea in infants, children, and international travelers, and accounts for more than 30% of diarrheal cases in children less than 5 years old. However, the choices of antimicrobial agents are now being limited by the ineffectiveness of many first-line drugs, in relation to the emergence of antimicrobial-resistant *E. coli* strains. The aim of this systematic review and meta-analysis was to provide an updated prevalence of antimicrobial-resistant DEC in Asia. A comprehensive systematic search was conducted on three electronic databases (PubMed, ScienceDirect, and Scopus), where 40 eligible studies published between 2010 and 2022 were identified. Using meta-analysis of proportions and a random-effects model, the pooled prevalence of DEC in Asian diarrheal patients was 22.8% (95% CI: 16.5–29.2). The overall prevalence of multidrug-resistant (MDR) and extended-spectrum beta-lactamase (ESBL)-producing DEC strains was estimated to be 66.3% (95% CI: 58.9–73.7) and 48.6% (95% CI: 35.1–62.1), respectively. Considering antimicrobial drugs for DEC, the resistance prevalence was highest for the penicillin class of antibiotics, where 80.9% of the DEC isolates were resistant to amoxicillin and 73.5% were resistant to ampicillin. In contrast, resistance to carbapenems such as imipenem (0.1%), ertapenem (2.6%), and meropenem (7.9%) was the lowest. The relatively high prevalence estimation signifies that the multidrug-resistant DEC is a public health threat. Effective antibiotic treatment strategies, which may lead to better outcomes for the control of *E. coli* infections in Asia, are necessary.

## 1. Introduction

*Escherichia coli* is a commensal bacterium that colonizes the intestinal tract and rarely causes disease in healthy individuals. However, some *E. coli* strains have evolved by acquiring virulence determinants via horizontal gene transfer [1,2]. These pathogenic strains are responsible for enteric diseases such as gastrointestinal and urinary tract infections in both healthy and immunocompromised hosts. *E. coli* strains involved in diarrheal diseases are known as diarrheagenic *E. coli* (DEC). DEC strains are classified into six pathotypes based on microbiological and epidemiological characteristics [3], as well as mechanisms of pathogenesis [4]. These six pathotypes include: (i) enterotoxigenic *E. coli* (ETEC), (ii) enteropathogenic *E. coli* (EPEC), (iii) enteroinvasive *E. coli* (EIEC), (iv) enterohemorrhagic *E. coli* (EHEC)—which is also known as Shiga-toxin-producing *E. coli* (STEC) or, less commonly, verotoxin-producing *E. coli* (VTEC)—(v) enteroaggregative *E. coli* (EAEC), and (vi) diffusely adherent *E. coli* (DAEC) [4,5,6].

DEC infections are typically associated with watery, mucous, or bloody diarrhea, and abdominal cramps, with or without fever [4,7]. Other clinical presentations may include nausea, vomiting, rapid pulse, decreased blood pressure, muscle cramps, and shock. Although most DEC infections are self-limiting, the progressive loss of bodily fluids and electrolytes may lead to severe dehydration and, eventually, death [7]. It has been estimated that more than 300 million cases of travel-associated diarrhea worldwide, with nearly 200,000 deaths, are caused by DEC. DEC strains—especially EAEC, EPEC, and ETEC—are responsible for around 30–40% of diarrhea episodes in children aged less than five years [8]. Diarrhea remains one of the leading causes of mortality in young children, causing more than 1.3 million deaths worldwide in 2013 [9]. The death toll among children increased to 1.6 million cases annually in 2016 [10]. In 2019, diarrhea accounted for approximately 9% of all deaths among children aged under 5 years globally, which translates to over 1300 young children dying each day [11]. Hence, appropriate treatment—such as maintenance of hydration and administration of antimicrobial therapy for severe and prolonged diarrhea—is important. However, the choices of antimicrobial agents are now limited by the ineffectiveness of many first-line drugs—such as ampicillin, ciprofloxacin, and sulfamethoxazole/trimethoprim [12]—in relation to the emerging antimicrobial-resistant strains resulting from the excessive use of antibiotics.

The global incidence of antimicrobial resistance in *E. coli* over the years is alarming and underlines the need for proper interventions to prevent transmission. Moreover, the emergence of multidrug-resistant (MDR) and extended-spectrum beta-lactamase (ESBL)-producing DEC strains has become a serious threat to public health, as the existing antibiotics are increasingly ineffective [13]. MDR and ESBL-producing DEC strains are superbugs that have the ability to resist to at least three different classes of antibiotics and to hydrolyze the third-generation cephalosporins—such as cefotaxime, ceftazidime, ceftriaxone, cefuroxime, and cefepime—or monobactams such as aztreonam [14]. The most common beta-lactamase (*bla*) genes reported in the ESBL-producing DEC isolates include *bla*_TEM_, *bla*_SHV_, and *bla*_CTX-M_, with the highest frequency of ESBL producers found in the EAEC pathotype [15,16,17]. The fact that these resistance genes are easily transferable among bacterial species via mobile genetic elements (e.g., plasmids, integrons, insertion sequences, and transposons) has made disease management for MDR and ESBL-producing DEC more challenging [18]. In the absence of new quality-assured antimicrobials, these groups of strains may cause untreatable infections and death. Hence, at present, updates on the antimicrobial resistance in DEC are essential for effective treatment to reduce the morbidity and mortality rates associated with diarrhea. This systematic review and meta-analysis (SRMA) was conducted to investigate the prevalence of antibiotic resistance in DEC strains in Asia from a decade of empirical published data.

## 2. Results

### 2.1. Study Selection and Characteristics of the Included Studies

**Figure 1:**
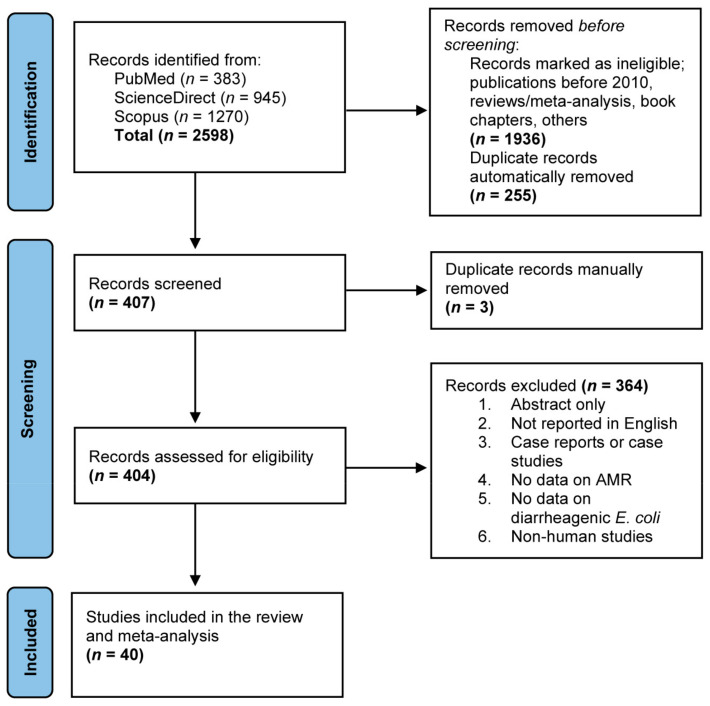
A PRISMA flow diagram of the study selection process. Three different electronic databases were utilized to search for eligible studies reporting antimicrobial-resistant diarrheagenic *Escherichia coli* (DEC) using defined search strategies. Records were combined and duplicates were removed using EndNote 20 software, followed by screening against predefined eligibility criteria before inclusion in the meta-analysis.

Figure 1 presents a Preferred Reporting Items for Systematic Reviews and Meta-analyses (PRISMA) flow diagram of the study selection process and the results of the literature search. Our search of three web databases returned a total of 2598 records, of which, following duplicate removal and pre-screening eligibility check, 407 records were screened for their title and abstract. After rounds of manual evaluation, a total of 404 records were retained for full-text assessment, where they were evaluated based on the predetermined exclusion criteria. An additional 364 articles were excluded for reasons that were not within the inclusion criteria. Consequently, a total of 40 articles were found to be eligible and, thus, were included in the qualitative study and meta-analysis. 

The detailed characteristics of the 40 included articles are presented in Table 1. Studies were carried out in 10 Asian countries, and China represented the highest number of DEC cases (*n* = 1592) from 10 different studies, although the majority of studies were conducted in Iran (*n* = 11). The second highest number of cases of DEC was in Bangladesh, which recorded 1180 cases from only three studies. Overall, a total of 5208 DEC cases were identified and subjected to antimicrobial susceptibility testing using 46 different antibiotics (Table 1). All selected studies were cross-sectional, conducted from 2000 to 2018, and published between 2011 and 2021. All bacterial species were isolated from stool specimens—collected mostly from pediatric groups suffering with diarrhea—and characterized using polymerase chain reaction (PCR) or double-disk synergy test (DDST) to detect the presence of ESBL genes. Biochemical tests such as the disk diffusion method (Kirby–Bauer method), agar dilution, and E-test were used in the antibiotic susceptibility tests. Of the 40 selected articles, 26 provided data on MDR, whereas 16 studies provided data on ESBL.

### 2.2. Prevalence of Diarrheagenic Escherichia coli

**Figure 2:**
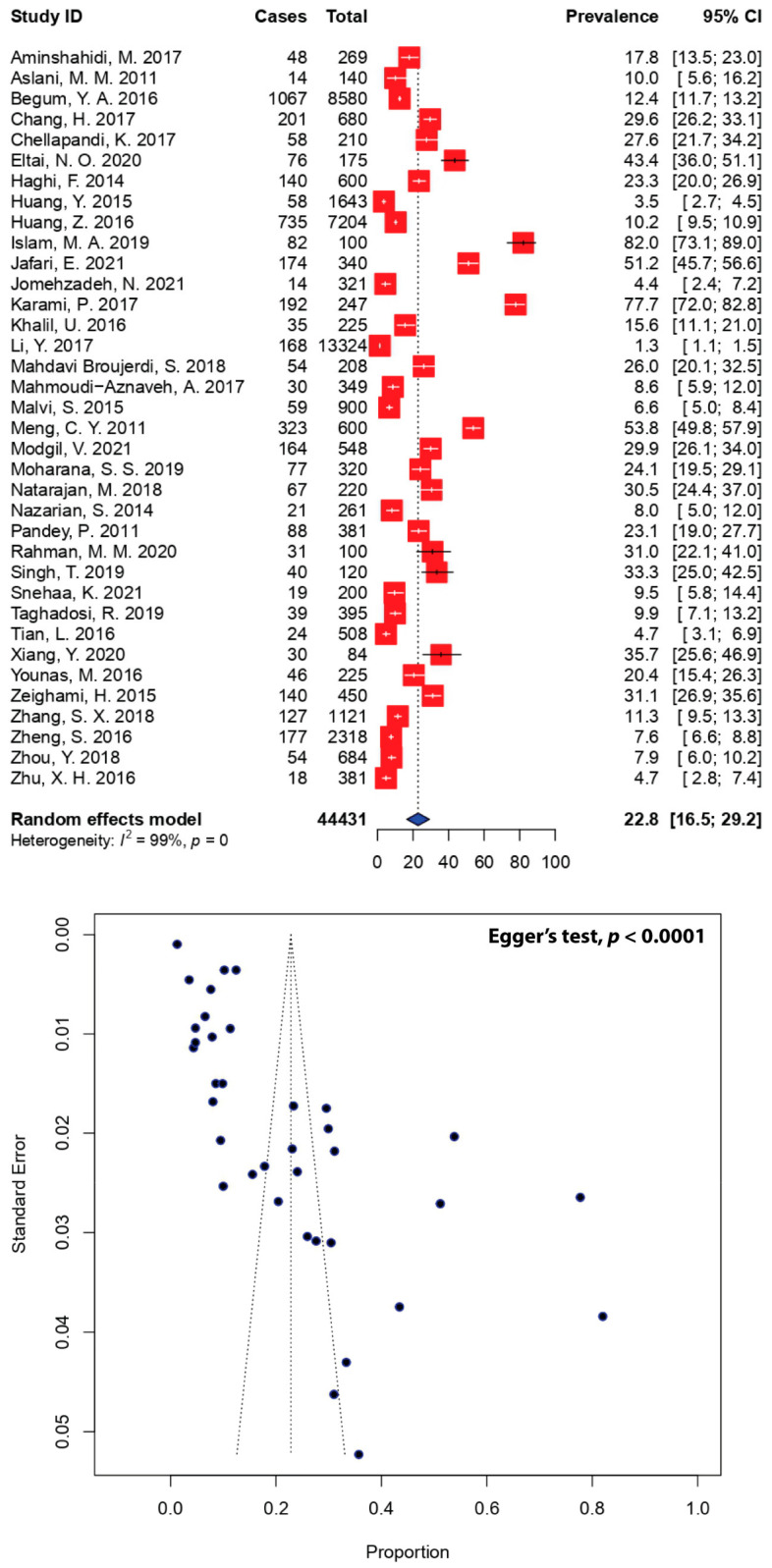
Forest and funnel plots representing the pooled Asian prevalence of DEC. The estimate of prevalence was calculated by pooling 36 selected studies using the random-effects model (top panel). Four studies (Bagus Wasito, E. 2017 [21], Hoang, P. H. 2017 [27], Margulieux, K. R. 2018 [18] and Pazhani, G. P. 2011 [44]) were excluded from the analysis due to the absence of data on the sample population. The distribution of effect estimates is shown by a funnel plot (bottom panel). Figures were generated using R software.

The pooled prevalence of DEC in Asian diarrheal patients from 36 studies was 22.8% (95% CI: 16.5–29.2) (Figure 2). The minimum and maximum prevalence of DEC was 1.3% and 82.0%, respectively. Four studies were excluded due to the absence of data on the sample population (Table 1). The study distribution, using a funnel plot, showed the asymmetrical distribution of effect estimates (Figure 2), which prompted us to further evaluate the data according to subgroups. Subgroup analysis was evaluated according to the regions and countries where the studies were conducted. When stratified by study region, the highest DEC prevalence of 53.8% (95% CI: 49.8–57.9) was recorded in Southeast Asia, which was represented by a single study in Cambodia. The lack of data from studies reported in Indonesia and Vietnam prompted us to exclude them from the analysis. Meanwhile, the lowest DEC prevalence of 11.1% (95% CI: 4.5–17.7) was recorded in East Asia. When stratified by country, the top three DEC prevalence estimates were recorded in Cambodia (53.8%, 95% CI: 49.8–57.9), Qatar (43.4%, 95% CI: 36.0–51.1), and Bangladesh (41.7%, 95% CI: 0.9–82.5), while China recorded the lowest DEC prevalence in Asia (11.1%, 95% CI: 4.5–17.7) (Table 2). Information regarding the gender of those infected with DEC was not reported in the majority of the included studies.

**Figure 3:**
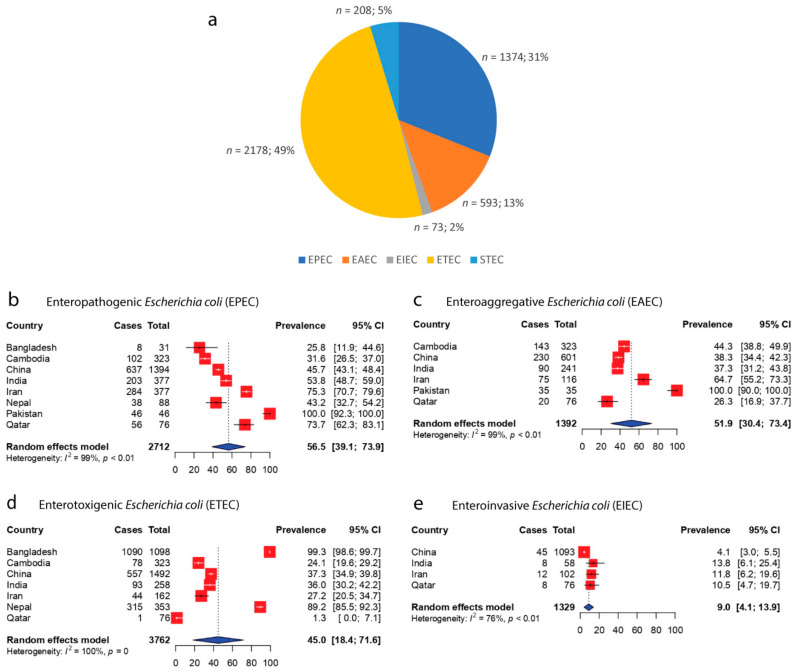
Pathotypes of DEC across Asian countries: (**a**) Proportions of different pathotypes of DEC. Enterotoxigenic *E. coli* constituted the highest proportion of DEC in Asia (*n* = 2178; 49%), followed by EPEC (*n* = 1374; 31%), EAEC (*n* = 593; 13%), STEC (*n* = 208; 5%), and EIEC (*n* = 73; 2%). (**b**) Forest plot representing the pooled Asian prevalence of enteropathogenic *E. coli* (EPEC). (**c**) Forest plot representing the pooled Asian prevalence of enteroaggregative *E. coli* (EAEC). (**d**) Forest plot representing the pooled Asian prevalence of enterotoxigenic *E. coli* (ETEC). (**e**) Forest plot representing the pooled Asian prevalence of enteroinvasive *E. coli* (EIEC).

When stratified according to the pathotypes of DEC, the highest number of isolates recorded were ETEC, which accounted for 49% of the 4426 total DEC cases reported (*n* = 2178), followed by EPEC (31%, *n* = 1374), EAEC (13%, *n* = 593), STEC (5%, *n* = 208), and EIEC (2%, *n* = 73) (Figure 3a). Only 4426 out of 5208 total DEC cases were characterized according to their pathotypes. Although ETEC constituted the largest proportion of DEC recorded in the included studies, the pooled prevalence of EPEC was the highest in Asia, at 56.5% (95% CI: 39.1–73.9), followed by EAEC (51.9%, 95% CI: 30.4–73.4), ETEC (45.0%, 95% CI: 18.4–71.6), and EIEC (9.0%, 95% CI: 4.1–13.9) (Figure 3). 

### 2.3. Antibiotic Resistance Patterns of Diarrheagenic Escherichia coli

In the 40 included studies, the antimicrobial susceptibility of the isolated DEC was tested against various antibiotics (Table 1). The pooled prevalence estimates of the resistant DEC isolates are presented in Table 3. In this study, the antibiotics were classified into 13 groups, with penicillin antibiotics being the most commonly used. In numerous studies conducted across Asia, ciprofloxacin has been identified as the most commonly used antibiotic (38 studies), followed by sulfamethoxazole/trimethoprim and gentamicin, while cefaclor and rifampicin were the least frequently tested (two studies each). The data generated in this meta-analysis revealed that resistant DEC strains exist for the majority of the antibiotics tested. The antibiotic resistance patterns for DEC isolated from Asian diarrheal patients revealed 80.2% (95% CI: 71.5–88.9) resistance to cefaclor, followed by ampicillin (80.9%; 95% CI: 71.1–90.3) and erythromycin (63.2%; 95% CI: 33.7–92.8), with meropenem (7.9%; 95% CI: 0.8–14.9), ertapenem (2.6%; 95% CI: 0.0–6.3), and imipenem (0.1%; 95% CI: 0.0–0.4) having the lowest DEC resistance rates. 

### 2.4. Prevalence of Multidrug-Resistant Diarrheagenic Escherichia coli

**Figure 4:**
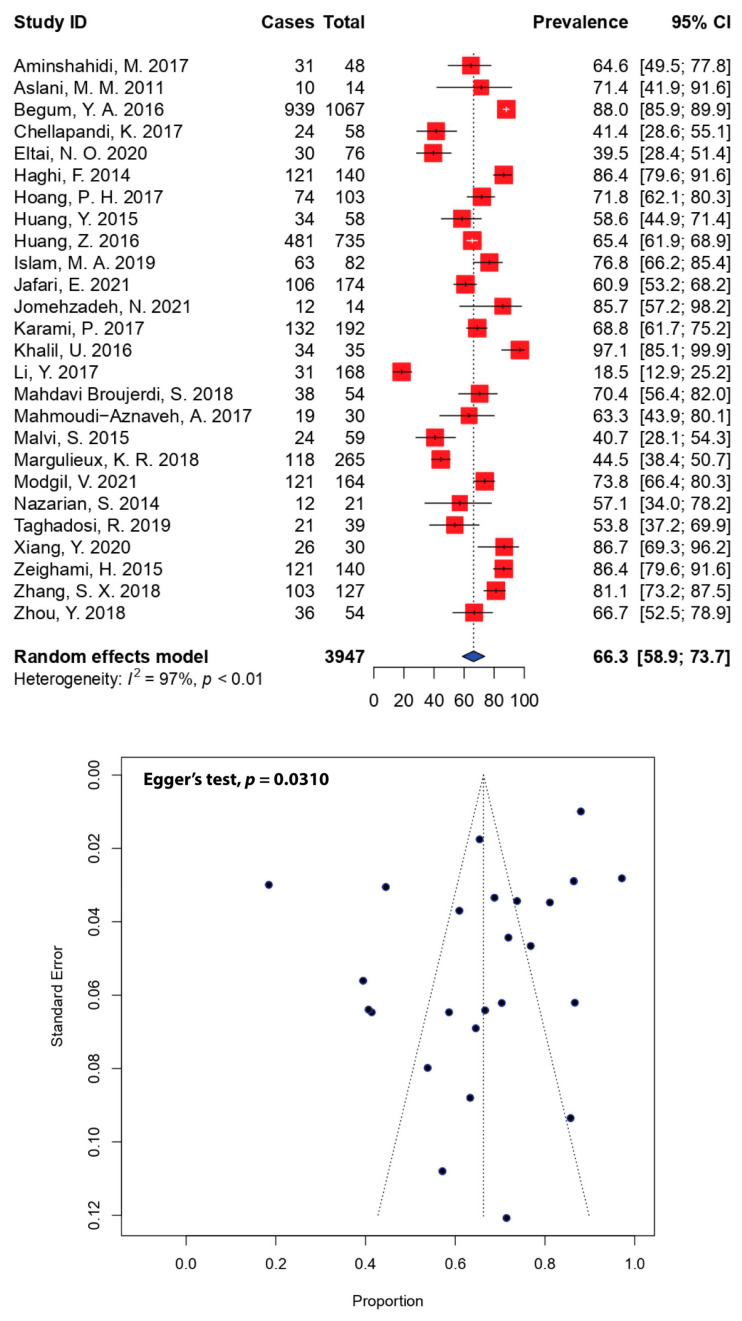
Forest and funnel plots representing the pooled prevalence of multidrug-resistant DEC in Asia. The estimate of prevalence was calculated by pooling 26 selected studies using the random-effects model (top panel). The distribution of effect estimates is shown by a funnel plot (bottom panel). Figures were generated using R software.

In this study, MDR was defined as resistance to three or more antimicrobial agents. The prevalence of MDR in DEC isolates was relatively high across individual studies, with 80.8% (21 out of 26) of the included studies reporting resistance rates higher than 50.0% of the total DEC isolates (Figure 4). The meta-analysis revealed that the pooled MDR-DEC prevalence was estimated to be 66.3% (95% CI: 58.9–73.7), with evidence of substantial heterogeneity rates (*I*^2^ = 97%, *p* < 0.01). The minimum and maximum prevalence of MDR-DEC was 18.5% [34] and 97.1% [33], respectively. The estimated prevalence of MDR was based on DEC isolates from 3947 human sources recruited in 26 studies over a 10-year period (2011–2021). The asymmetrical funnel plot (Figure 4) indicates the presence of publication bias, which was statistically confirmed by Egger’s test (*p* = 0.0310).

### 2.5. Patterns of Extended-Spectrum-β-lactamase-Producing Escherichia coli

**Figure 5:**
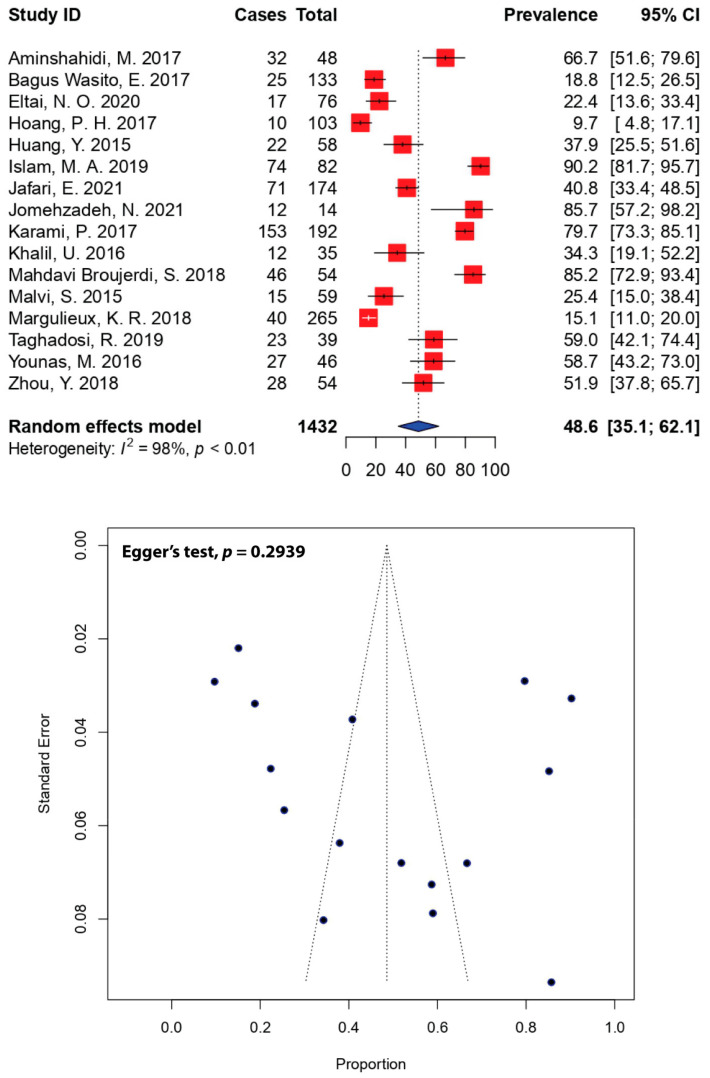
Forest and funnel plots representing the pooled prevalence of extended-spectrum β-lactamase (ESBL)-producing DEC in Asia. The estimate of prevalence was calculated by pooling 16 selected studies using the random-effects model (top panel). The distribution of effect estimates is shown by a funnel plot (bottom panel). Figures were generated using R software.

Of the 40 eligible studies, 16 studies with a total sample size of 1432 reported ESBL-producing DEC in Asian diarrheal patients. After pooling the results of these studies, the overall prevalence of ESBL-producing DEC in Asia was estimated to be 48.6% (95% CI: 35.1–62.1), with significant heterogeneity between studies (*I*^2^ = 98%, *p* < 0.01). As shown in Figure 5, the highest prevalence of ESBL-producing DEC was reported in a study conducted in Bangladesh (90.2%, 95% CI: 81.7–95.7) [30], while the lowest was reported in a study conducted in Vietnam (9.7%, 95% CI: 4.8–17.1) [27].

## 3. Discussion

Diarrhea remains one of the leading causes of mortality in young children, being responsible for more than 1.6 million deaths worldwide in 2016 [10], while in 2019 it accounted for approximately 9% of all deaths among young children, including over 1300 children under the age of 5 years dying each day [11]. In a more recent global disease burden report published in 2020, diarrheal diseases ranked 3rd among the top 10 causes of death in children aged less than 9 years [56]. Moreover, more than 25% of deaths caused by diarrhea occurred in young children less than 5 years old, of which about 90% of were reported in sub-Saharan Africa and South Asia [57]. DEC—particularly EPEC and ETEC producing heat-stable toxins—is among the few pathogens attributed to moderate-to-severe cases of diarrhea [58]. However, the global emergence of drug-resistant strains, limiting the choice of effective antimicrobial drugs for diarrhea treatment, has become the main challenge in the treatment of DEC infections. For instance, in recent years, ETEC has been reported to be becoming resistant to many first-line drugs, such as ampicillin, nalidixic acid, tetracycline, sulfonamides, and azithromycin [34,50]. This phenomenon can be seen not only in Asia, but also in other regions [22,59,60,61]. Thus, determining their burden in a population is crucial to the design of targeted strategies to reduce the incidence of mortality from diarrhea. This requires comprehensive data on the prevalence and patterns of antimicrobial-resistant DEC but, to the best of our knowledge, no such study is available to date in Asia.

Our findings in this SRMA were calculated by combining all eligible data on the prevalence of antimicrobial-resistant DEC from hospital- and community-based studies, as reported in the 40 selected studies in Asia. Nonetheless, as expected from different studies with various backgrounds and settings, the outcomes were mostly heterogeneous. The variability in our data was most likely due to the fact that different methodological variations, sample sizes, and research settings—such as study period, regions, and population age—were utilized in different studies. This is expected, as our SRMA used random-effects meta-analysis, which presumes heterogeneity, as opposed to meta-analysis, which uses a fixed-effects model [62]. Nevertheless, our SRMA is the first to evaluate the prevalence of antibiotic-resistant DEC in Asia and will hopefully be useful for designing targeted strategies.

The pooled estimate revealed that 22.8% (95% CI: 16.5–29.2) of all diarrheal cases in Asia in the last two decades were caused by DEC (Figure 2), where the minimum and maximum prevalence of DEC was 1.3% [34] and 82.0% [30], respectively. The majority of the studies reporting the prevalence of DEC analyzed in this SRMA were from Iran, accounting for 11 different studies from a total of 40. This is to be expected, as DEC infections have been a major concern in Iran for the past few decades. The pooled prevalence of DEC in Iran was estimated at 24.3% (95% CI: 11.1–37.5)—slightly above the pooled estimate of DEC in Asia in general (Table 2). The prevalence of DEC identified in the present study was higher than the findings of a similar comprehensive estimate from Iran, which was previously estimated at 17.0% (95% CI: 16.9–18.0; *I*^2^ = 98.9, *p* < 0.001) [63]. Differences in prevalence estimates between the two studies might be attributed to the variations in sample size, the number of included studies, publication year, study settings, and many other factors. It should be noted that the prevalence of DEC in this study was higher, possibly due to the smaller number of included studies, all of which were published after 2010, whereas there were 73 included studies published since 1990 in the previous SRMA, of which 14 were written in the Persian language [63]. Despite fewer studies having been included in this SRMA, our pooled estimates represent the current prevalence of DEC in the country.

Conversely, the pooled estimate of DEC in China was 11.1% (95% CI: 4.5–17.7)—lower than the prevalence of DEC in Asia in general (Table 2). The majority of the included studies from China had lower pooled estimates of DEC, with the minimum and maximum prevalence of DEC in the country being 1.3% [34] and 29.6% [23], respectively. It is important to note that China had the largest sample population of 27,947 from 10 included studies that constituted 62.9% of the total diarrheal Asian population analyzed in this SRMA. In contrast, Cambodia and Qatar had the highest prevalence of DEC, estimated at 53.8% (95% CI: 49.8–57.9) [38] and 43.4% (95% CI: 36.0–51.1) [25], respectively, each from a single study. While larger sample populations contributed to lower pooled estimates in China, the smaller sample populations from Qatar and Cambodia resulted in higher DEC prevalence in both countries. 

Concerning the antimicrobial drugs for the treatment of DEC, the Centers for Disease Control and Prevention (CDC) Yellow Book 2020 recommends that antibiotics used to treat non-STEC DEC include fluoroquinolones such as ciprofloxacin, macrolides such as azithromycin, and rifaximin (or other rifamycin derivatives, such as rifampicin), although they are not recommended for the treatment of STEC due to the possible risk of hemolytic uremic syndrome [64]. In this meta-analysis, resistance was common among DEC isolates, particularly against the penicillin class of antibiotics. The pooled prevalence of resistant DEC against amoxicillin was the highest, recorded at 80.9% (95% CI: 71.5–90.3), whereas it was 73.5% (95% CI: 67.1–79.8) against ampicillin (Table 3). While the prevalence of resistant DEC was high against the penicillin antibiotics, combinations with other antimicrobial agents somehow increased the susceptibility of DEC to amoxicillin and ampicillin. For instance, the combination of amoxicillin with clavulanic acid rendered DEC more susceptible to the antibiotic, with the pooled prevalence of resistant DEC decreased to 34.5% (95% CI: 20.9–48.1). Similarly, the combination of ampicillin with sulbactam, on the other hand, decreased the resistance of DEC from 73.5% to 22.6% (95% CI: 8.1–37.2). Additionally, the use of piperacillin in the treatment of DEC recorded 47.7% (95% CI: 14.0–81.5) resistance, while the combination of piperacillin with tazobactam reduced the prevalence substantially to 8.4% (95% CI: 3.0–13.8). It is possible that utilizing more than one β-lactam inhibitor in the treatment of DEC increases the susceptibility of the pathogen significantly. High rates of resistance to amoxicillin, ampicillin, and piperacillin are unfortunate events in the majority of Asian countries, and could reflect the excessive and unjustified use of antibiotics in general care.

High proportions of DEC isolates were also resistant against fluoroquinolones such as ciprofloxacin (34.5%, 95% CI: 20.9–48.1), levofloxacin (80.9%, 95% CI: 71.5–90.3), norfloxacin (22.6%, 95% CI: 8.1–37.2), and ofloxacin (47.7%, 95% CI: 14.0–81.5), as well as the synthetic quinolone antibiotic nalidixic acid (73.5%, 95% CI: 67.1–79.8), in spite of their importance as first-line treatments for diarrhea. Additionally, high resistance against macrolides such as azithromycin (38.9%, 95% CI: 18.4-59.3) and erythromycin (63.2%, 95% CI: 33.7–92.8), rifampicin (48.8%, 95% CI: 0.0–100.0), tetracycline (54.7%, 95% CI: 46.9–62.6), and sulfamethoxazole/trimethoprim (50.0%, 95% CI: 42.0–58.0) was alarming, as choices of antimicrobial agents are now limited by the ineffectiveness of many antibiotics. Resistance of *E. coli* against a wide array of antimicrobials was also reported by a recent comprehensive study in Ethiopia, where 78.0%, 76.9%, and 67.0% of *E. coli* isolates were resistant to ampicillin, tetracycline, and sulfamethoxazole/trimethoprim, respectively [65]. In another report, high rates of resistance to amoxicillin (70.5%) and tetracycline (54.6%) were also observed in *E. coli* isolates [66]. Nevertheless, our results showed that resistance to carbapenems such as ertapenem (2.6%, 95% CI: 0.0–6.3), imipenem (0.1%, 95% CI: 0.0–0.4), and meropenem (7.9%, 95% CI: 0.8–14.9) was the lowest among the tested antibiotics. Although the resistance rates against these antibiotics were low, careful and justified use of such antibiotics is necessary to limit the emergence of more drug-resistant strains. 

Moreover, the high resistance burden of many first-line drugs has resulted in relatively high rates of MDR in DEC isolates. The pooled estimate of MDR-DEC in Asia was 66.3% (95% CI: 58.9–73.7) (Figure 4)—substantially higher than other reports from Nigeria (50.0%) [67] and Spain (40.0%) [68], but lower than a report from Ethiopia (78.2%) [65]. Our pooled estimate was much higher than previous estimates among human isolates globally (22%) [66] and from community settings in low- and middle-income countries (28%) [69]. The majority of the included studies recorded high prevalence of MDR, where the minimum and maximum prevalence of MDR-DEC in Asia was 18.5% [34] and 97.1% [33], respectively. This is worrying, as resistance to multiple antimicrobials in the population has steadily been increasing in the past decades, especially in relation to resistance against quinolones and third-generation cephalosporins [70]. Multidrug-resistant pathogens are currently considered to be a global threat and a major problem for public health. For instance, among diarrheagenic pathogens in Ethiopia, MDR *Campylobacter* recorded 80.8% prevalence, while the pooled estimates of MDR *Shigella* and *Salmonella* were 79.0% and 59.5%, respectively [65]. In Spain, the prevalence of MDR *Klebsiella pneumoniae* was recorded at 32.5%, whereas the prevalence of MDR *Acinetobacter baumannii* and *Pseudomonas aeruginosa* was 10.0% and 5.0%, respectively [68]. Although relatively low, the data did not represent the collective prevalence of the MDR-DEC pathogens in the country, due to the limited target population and study methods (single study versus pooled meta-analysis reports).

Resistance in non-pathogenic *E. coli* strains may not endanger health in healthy individuals; however, non-pathogenic strains that have acquired virulence genes may induce disease that may not be easily treated. They could also act as a reserve for the acquisition of virulence determinants for other bacterial species [37]. Moreover, the acquisition of ESBL genes is one of the main mechanisms that could lead to an increase in MDR-DEC. In our meta-analysis, the prevalence of ESBL-producing DEC in Asia was 48.6% (95% CI: 35.1–62.1). In the United States, the prevalence of ESBL-producing *E. coli* showed a significantly increasing trend, reported at 7.8% and 18.3% in 2010 and 2014, respectively [71]. In a comprehensive study of the European population, the prevalence of ESBL-producing Enterobacteriaceae in long-term care facilities was reported to be 10–60% [72]. ESBL-producing *E. coli* have been shown to have good susceptibility to carbapenems and amikacin [21,73,74]; this is largely consistent with our findings, which showed good susceptibility to carbapenems and amikacin (Table 3). 

Our SRMA is the first to provide a comprehensive estimation of antimicrobial-resistant DEC in Asia. Nonetheless, our analysis has several limitations. The first and most important limitation is that the included studies (*n* = 40) did not incorporate all of the countries in Asia; thus, the calculated prevalence might not fully represent the true proportions of antimicrobial-resistant DEC in Asia. However, a significant number of studies were included from 10 Asian countries; thus, data from a large number of participants (*n* = 47,476) were analyzed. Second, although it is common in meta-analyses of prevalence estimation [63,65,66,72,75], significant heterogeneity was observed in the included studies. This was expected, as a random-effects model was used in our meta-analysis, which presumes heterogeneity [62]. Third, we were unable to account for the potential effects of age and gender distribution on the prevalence of antimicrobial-resistant DEC, due to the nature of data presentation in many of the included studies. Although some studies reported antimicrobial resistance data from pediatric patients aged less than 5 years, some others reported pediatric data from different age groups, while some reported data from various age groups. In addition, patterns of multidrug resistance of different DEC strains also could not be obtained, owing to the different data presented in various studies. The majority of the included studies did not report the prevalence of antimicrobial resistance in specific DEC strains, but instead reported the resistance of DEC collectively. We believe that such vital information would be helpful to researchers, clinicians, and governments.

## 4. Methodology

### 4.1. Literature Search Strategy and Selection

This study was performed based on the PRISMA guidelines [76]. The protocol of this study was registered on the International Prospective Register of Systematic Reviews (PROSPERO) database (registration number: CRD42022349666). A comprehensive literature search was conducted from March 2022 to May 2022 to find selected studies on the prevalence of antimicrobial-resistant diarrheagenic *E. coli* (DEC) in Asia that were available in the PubMed, ScienceDirect, and Scopus databases (Figure 1). Relevant terms and keywords were used to retrieve all relevant articles from the databases, including “*Escherichia coli* AND diarrheagenic AND drug resistance AND Asia”, “*Escherichia coli* AND Shiga toxin-producing AND drug resistance AND Asia”, “*Escherichia coli* AND enteropathogenic AND drug resistance AND Asia”, “*Escherichia coli* AND enteroaggregative AND drug resistance AND Asia”, “*Escherichia coli* AND enteroinvasive AND drug resistance AND Asia”, and “*Escherichia coli* AND enterotoxigenic AND drug resistance AND Asia”. Additionally, the reference lists of the selected articles were also screened to increase the chance of obtaining more articles. All articles that fulfilled the selection criteria were used in the analysis.

### 4.2. Inclusion and Exclusion Criteria 

Studies that reported adequate data for calculating the prevalence of antimicrobial resistance in diarrheal patients from all countries in Asia, regardless of age and gender, were considered eligible for inclusion in this study. Only full-length published original research articles in the English language were included in the analysis. In order to obtain updated information on the topic, only articles published from 2010 to March 2022 were considered. In contrast, studies that did not report data from diarrheal patients or their antimicrobial susceptibility, as well as review articles, case studies and case reports, and studies with abstracts only, were excluded from the analysis. All articles published before 2010 and unpublished or incomplete information were not included in the study. Only data from human-related studies were included.

### 4.3. Data Extraction and Quality Control

All selected studies were retrieved and managed using the EndNote 20 reference management software, where duplicates were removed, and the remaining articles were examined thoroughly based on their titles and abstracts. Full texts were then further assessed and examined for the determination of eligible studies. Mohd Zulkifli Salleh (M.Z.S.) and Nik Mohd Noor Nik Zuraina (N.M.N.N.Z.) independently evaluated the eligibility of all articles using predetermined inclusion criteria. The selected articles were then coded, and data were extracted in a table in Microsoft Excel, consisting of the author’s name, title, year of publication, study period, study region, study design, sample population, sample size, sample type, age, isolated bacterial species, resistance patterns of the isolates, and prevalence of MDR and ESBL-producing DEC.

### 4.4. Data Analysis

The meta-analysis was performed using metaprop codes in the meta (version 5.2-0) and metafor (version 3.4-0) packages of R (version 4.2.1), as implemented in RStudio (version 2022.02.2+485) [77]. The pooled prevalence of resistance to any antimicrobials, MDR, ESBL, and 95% confidence interval (CI) were calculated using the REML method for the random-effects model. Statistical heterogeneity between studies was evaluated using the Cochran’s Q test for the significance of heterogeneity and the inconsistency index (*I*^2^) [78], where *I*^2^ > 75% and a significance level < 0.05 (*p*-value) were deemed to represent substantial heterogeneity. In addition, publication bias was calculated by evaluating a funnel plot, which was tested for significance with Egger’s test only for the included studies of more than 10 subjects.

## 5. Conclusions

Our SRMA provides evidence of a substantial distribution of antimicrobial-resistant DEC in Asia. The pooled estimates demonstrated a significantly high prevalence of DEC in Asia, posing a major new problem for public health. Our analysis revealed that the pooled prevalence of MDR and ESBL-producing DEC in Asia was 66.3% and 48.6%, respectively. While the prevalence varied across different countries in Asia, the evidence signifies that multidrug resistance is a public health threat worthy of careful consideration. It is therefore crucial to continuously monitor drug-resistant DEC by performing robust drug susceptibility tests as well as establishing an antimicrobial surveillance system so that reliable antibiotic strategies can be implemented, which may lead to better outcomes for the control and treatment of *E. coli* infections in Asia as well as in different parts of the world.

## Figures and Tables

**Table 1:** Major characteristics of the 40 included studies in this systematic review and meta-analysis (SRMA).

No	Study ID (Author, Year)	Ref.	Study Period	Country	Sample Population	Age	Sample Size	Bacterial Species	Study Methods	Tested Antibiotics
1	Aminshahidi, M. 2017	[19]	8/2014 to 2/2015	Iran	269	0 to 18 years	48	EAEC, EIEC, EPEC, ETEC	KB, PCR	MEM, CAZ, CTX, CTR, CIP, AMK, AMP, SXT, GEN
2	Aslani, M. M. 2011	[20]	7/2007 to 5/2008	Iran	140	0 to 12 years	14	EAEC	KB, PCR	AMP, ERY, CEP, SXT, TET, NAL, CFM, AMC, CTX, CTR, CHL, GEN, CIP, NOR
3	Bagus Wasito, E. 2017	[21]	2012	Indonesia	ND	0 to 3 years	133	DEC	KB, DDST, PCR	GEN, AMK, SXT, CIP, TET
4	Begum, Y. A. 2016	[22]	2005 to 2009	Bangladesh	8580	ND	1067	ETEC	KB, PCR	AMP, AZI, CIP, CTR, SXT, DOX, ERY, NAL, NOR, STR, TET
5	Chang, H. 2017	[23]	2014	China	680	Pediatrics	201	EPEC, EAEC, ETEC, STEC	KB, PCR	CHL, GEN, NAL, CIP, SXT, TET, AMP, AMC, CTR, FOX, AZI
6	Chellapandi, K. 2017	[24]	11/2013 to 10/2015	India	210	0 to 5 years	58	EPEC, EIEC	KB, PCR	AMP, AMK, CHL, CTR, LEX, CIP, SXT, CFP, MEM, NOR, GEN, CFM, DOX, OFX
7	Eltai, N. O. 2020	[25]	8/2017 to 1/2018	Qatar	175	0 to 10 years	76	EPEC, EAEC, EIEC, ETEC	E-test, PCR	AMP, AMC, TZP, GEN, CIP, CHL, TET, SXT, CEP, CFX, CTR, CEF
8	Haghi, F. 2014	[26]	3/2011 to 1/2012	Iran	600	0 to 5 years	140	DEC	KB, PCR	AMX, AZT, AMK, FOX, CTX, CAZ, AMC, SXT, CIP, GEN, ERY, IMI, TET
9	Hoang, P. H. 2017	[27]	3/2013 to 11/2013	Vietnam	ND	20 to 70 years	103	DEC	KB, DDST, PCR	AMP, CTX, CAZ, IMI, NAL, CIP, GEN, KAN, STR, FOS, TET, SXT, CHL
10	Huang, Y. 2015	[28]	2009	China	1643	Pediatrics	58	EPEC	KB, PCR	AMP, SXT, CHL, CIP, CTR, CAZ, IMI
11	Huang, Z. 2016	[29]	6/2012 to 10/2013	China	7204	All group	735	EPEC, ETEC, EIEC, STEC	Agar dilution, PCR	STR, AMP, NAL, SUL, TET, TMP, SXT, AMC, CTX, CEF, GEN, CAZ, CHL, CIP, OFX, IMI
12	Islam, M. A. 2019	[30]	3/2017 to 10/2017	Bangladesh	100	0 to 1 year	82	DEC	KB, DDST, PCR	AMP, AZI, CEF, NAL, CIP, SXT, TET, GEN, CHL, MEM, IMI, COL, NIT
13	Jafari, E. 2021	[31]	2014	Iran	340	5 months to 92 years	174	STEC	KB, PCR	CTX, CAZ, TZP, ETP, IMI, CIP, LEV, AMK, TET, AMP, SXT
14	Jomehzadeh, N. 2021	[15]	3/2016 to 2/2017	Iran	321	0 to 5 years	14	EPEC	KB, DDST, PCR	CAZ, CTR, CTX, FOX, CIP, IMI, MEM, SXT, AMK, TET, GEN, CHL, AMX, PIP, AZT
15	Karami, P. 2017	[32]	Summer months	Iran	247	0 to 10 years	192	EPEC	KB, DDST, PCR	AMP, FAM, CAZ, CTX, CTR, IMI, AZT, GEN, AMK, TET, CIP, TMP, CHL
16	Khalil, U. 2016	[33]	7/2010 to 8/2011	Pakistan	225	0 to 5 years	35	EAEC	KB, DDST, PCR	TET, NAL, SXT, ERY, GEN, DOX, CEC, CHL, CIP, AMP
17	Li, Y. 2017	[34]	2009 to 2014	China	13324	20 to 59 years	168	ETEC	KB	NAL, CEP, AMP, TET, STR, TMP, SXT, AMC, CTX, CTR, GEN, FAM, CEF, KAN, CIP, LEV, CAZ, FOX, AMK, CHL, IMI, MEM
18	Mahdavi Broujerdi, S. 2018	[35]	9/2015 to 6/2016	Iran	208	0 to 5 years	54	EAEC, EPEC, EIEC, ETEC	KB, DDST, PCR	CTR, CIP, AMK, GEN, FOX, CAZ, CTX, IMI
19	Mahmoudi-Aznaveh, A. 2017	[36]	11/2012 to 10/2013	Iran	349	0 to 5 years	30	EPEC	KB, PCR	TET, SXT, NAL, STR, MIN, GEN, CIP
20	Malvi, S. 2015	[37]	2012 to 2013	India	900	0 to 10 years	59	EPEC	KB, DDST, PCR	AMX, CHL, SXT, NAL, CIP, NOR, GEN, AMK, CTR, CEF, IMI, MEM, ETP, TZP, AZI
21	Margulieux, K. R. 2018	[18]	2001 to 2016	Nepal	ND	ND	265	ETEC	KB, DDST, PCR	AMP, SXT, TET, FAM, CIP, ETP
22	Meng, C. Y. 2011	[38]	11/2004 to 10/2006	Cambodia	600	3 months to 5 years	323	ETEC, EPEC, EAEC	KB	AZI, ERY, NAL, CIP, AMP, GEN, SXT, TET
23	Modgil, V. 2021	[39]	2015 to 2017	India	548	0 to 5 years	164	EAEC, EPEC, ETEC	KB, PCR	AMP, CIP, AMK, IMI, LEV, GEN, CFM, TZP, ETP, SXT, FOX, CTR
24	Moharana, S. S. 2019	[40]	9/2015 to 11/2017	India	320	0 to 5 years	77	ETEC, EPEC, EAEC	KB, DDST, PCR	AMK, SXT, IMI, NAL, OFX, CTR, AMP, LEV, AMC, CIP
25	Natarajan, M. 2018	[41]	7/2015 to 6/2016	India	220	All groups	67	DEC	KB, PCR	CTR, CAZ, SXT, TET, LEV, CIP, GEN, AMK, CHL
26	Nazarian, S. 2014	[42]	4/2010 to 9/2011	Iran	261	0 to 5 years	21	ETEC	KB, PCR	AMP, NAL, CHL, TET, CIP, GEN, FOX, CEP, SXT
27	Pandey, P. 2011	[43]	3/2001 to 3/2003	Nepal	381	>18 years	88	ETEC, EPEC	KB	AMX, SXT, NAL, CIP, AZI
28	Pazhani, G. P. 2011	[44]	2000	India	ND	ND	17	ETEC	KB, PCR	AMP, CEP, TET, SXT, NAL, NOR, CIP, CHL, STR, KAN
29	Rahman, M. M. 2020	[45]	3/2018 to 5/2018	Bangladesh	100	ND	31	ETEC, EPEC	KB, PCR	AMC, TET, NAL, AZI, CIP, AMP, ERY
30	Singh, T. 2019	[46]	7/2013 to 7/2015	India	120	0 to 5 years	40	DEC	KB, PCR	NOR, CTX, IMI, MEM, CAZ, AZT, NAL, AMC, GEN, CIP, AMP, AMK, PLB, CTR, TZP
31	Snehaa, K. 2021	[47]	6/2014 to 6/2015	India	200	0 to 5 years	19	EPEC	KB, PCR	NAL, CTX, AMK, GEN, TZP, IMI, AZT, NOR
32	Taghadosi, R. 2019	[48]	10/2014 to 11/2015	Iran	395	All groups	39	EPEC, ETEC	KB, DDST, PCR	AMC, AZT, PIP, CIP, CTR, KAN, IMI, AMP, NAL, AMK, SXT, CAZ, CFX, TOB
33	Tian, L. 2016	[49]	5/2014 to 8/2015	China	508	0 to 5 years	24	EPEC, STEC, EAEC	Agar dilution, PCR	AMP, FAM, CTR, CTX, NAL, CIP, LEV, SXT, AZI, CHL, TET, CFZ, CFX, IMI, AMK, GEN, ERY, DOX
34	Xiang, Y. 2020	[50]	2016 to 2018	China	84	20 to 60 years	30	ETEC	Agar dilution	CTR, TET, TIO, FOX, GEN, AMP, CHL, CIP, SXT, SUL, NAL, STR, AZI, AMC
35	Younas, M. 2016	[51]	2010 to 2012	Pakistan	225	0 to 5 years	46	EPEC	KB, DDST, PCR	AMP, CEC, ERY, AMC, SXT, TET, NAL, CIP, GEN
36	Zeighami, H. 2015	[52]	3/2011 to 1/2012	Iran	450	0 to 5 years	140	DEC	KB, PCR	AMX, AZT, AMK, FOX, CTX, CAZ, AMC, SXT, CIP, GEN, IMI, TET
37	Zhang, S. X. 2018	[53]	6/2014 to 7/2015	China	1121	All groups	127	DEC	KB	AMP, AMC, CEP, CIP, CTX, GEN, NAL, RIF, SXT, TET
38	Zheng, S. 2016	[54]	7/2009 to 12/2014	China	2318	0 to 5 years	177	EAEC, EPEC, ETEC, STEC, EIEC	KB, PCR	AMP, TET, CFZ, SXT, PIP, CFX, FAM, CTX, GEN, AZT, CIP, CAZ, AMC, CEF, FOX, TZP, AMK, IMI, MEM
39	Zhou, Y. 2018	[16]	8/2015 to 9/2016	China	684	0 to 5 years	54	EPEC, EAEC, ETEC, EIEC, STEC	Agar dilution, PCR	MEM, IMI, TZP, SXT, AMK, GEN, LEV, CIP, FOX, AZT, CEF, CAZ, CTX, CFX, CFZ, AMP
40	Zhu, X. H. 2016	[55]	7/2014 to 6/2015	China	381	0 to 5 years	18	EAEC, EPEC, STEC	Agar dilution	AMP, FAM, CFZ, CFX, CTX, IMI, AMK, GEN, CIP, LEV, CHL, TET, SXT

ND, no data; DEC, diarrheagenic *Escherichia coli*; EAEC, enteroaggregative *Escherichia coli*; EIEC, enteroinvasive *Escherichia coli*; ETEC, enterotoxigenic *Escherichia coli*; EPEC, enteropathogenic *Escherichia coli*; STEC, Shiga-toxin-producing *Escherichia coli*; KB, Kirby–Bauer disk diffusion; DDST, double-disk synergy test; PCR, polymerase chain reaction; AMC, amoxicillin and clavulanic acid; AMK, amikacin; AMP, ampicillin; AMX, amoxicillin; AZI, azithromycin; AZT, aztreonam; CAZ, ceftazidime; CEC, cefaclor; CEF, cefepime; CEP, cephalothin; CFM, cefixime; CFP, cefoperazone; CFX, cefuroxime; CFZ, cefazolin; CHL, chloramphenicol; CIP, ciprofloxacin; COL, colistin; CTR, ceftriaxone; CTX, cefotaxime; DOX, doxycycline; ETP, ertapenem; ERY, erythromycin; FAM, ampicillin and sulbactam; FOS, fosfomycin; FOX, cefoxitin; GEN, gentamicin; IMI, imipenem; KAN, kanamycin; LEV, levofloxacin; LEX, cefalexin; MEM, meropenem; MIN, minocycline; NAL, nalidixic acid; NIT, nitrofurantoin; NOR, norfloxacin; OFX, ofloxacin; PIP, piperacillin; PLB, polymyxin B; RIF, rifampicin; STR, streptomycin; SXT, sulfamethoxazole and trimethoprim; TET, tetracycline; TIO, ceftiofur; TMP, trimethoprim; TOB, tobramycin; TZP, piperacillin and tazobactam.

**Table 2:** Pooled prevalence of DEC in different Asian regions.

Subgroup	Prevalence (%) [95% Cis]	No. Of Studies	Sample Size	Sample Population	*I* ^2^	*p*-Value
*Regions*						
East Asia	11.1 [4.5–17.7]	10	1592	27947	99%	<0.01
Middle East	25.9 [13.4–38.3]	12	942	3755	99%	<0.01
South Asia	26.4 [16.3–36.5]	13	1833	12129	98%	<0.01
Southeast Asia	53.8 [49.8–57.9]	1	323	600	NA	NA
*Countries*						
Bangladesh	41.7 [0.9–82.5]	3	1180	8780	99%	<0.01
Cambodia	53.8 [49.8–57.9]	1	323	600	NA	NA
China	11.1 [4.5–17.7]	10	1592	27947	99%	<0.01
India	22.7 [14.7–30.8]	7	484	2518	97%	<0.01
Iran	24.3 [11.1–37.5]	11	866	3580	99%	<0.01
Nepal	23.1 [19.0–27.7]	1	88	381	NA	NA
Pakistan	17.9 [13.1–22.6]	2	81	450	45%	0.18
Qatar	43.4 [36.0–51.1]	1	76	175	NA	NA

**Table 3:** Pooled prevalence of antimicrobial-drug-resistant DEC in Asia.

Antibiotics	Prevalence (%) [95% CIs]	No. of Resistant Isolates	No. of Studies	*I* ^2^	*p*-Value
***1st gen. Cephalosporins***					
Cefaclor (CEC)	80.2 [71.6–88.9]	65	2	0%	0.96
Cephalothin (CEP)	48.4 [24.2–72.5]	161	6	96%	<0.01
Cefazolin (CFZ)	48.3 [34.5–62.0]	148	4	69%	0.02
***2nd gen. Cephalosporins***					
Cefuroxime (CFX)	42.5 [29.9–55.0]	168	6	84%	<0.01
Cefoxitin (FOX)	17.6 [7.0–28.3]	177	9	96%	<0.01
***3rd gen. Cephalosporins***					
Ceftazidime (CAZ)	29.5 [17.2–41.9]	557	17	99%	<0.01
Ceftriaxone (CTR)	31.7 [19.7–43.7]	617	20	98%	<0.01
Cefotaxime (CTX)	36.6 [27.4–45.7]	720	18	96%	<0.01
***4th gen. Cephalosporins***					
Cefepime (CEF)	26.3 [8.4–44.3]	303	7	98%	<0.01
***Aminoglycosides***					
Amikacin (AMK)	10.9 [6.5–15.4]	169	21	89%	<0.01
Gentamicin (GEN)	21.3 [16.4–26.1]	675	31	92%	<0.01
Kanamycin (KAN)	14.7 [0.0–29.9]	35	5	91%	<0.01
Streptomycin (STR)	48.7 [25.7–71.8]	1317	7	99%	<0.01
***Carbapenems***					
Ertapenem (ETP)	2.6 [0.0–6.3]	14	4	79%	<0.01
Imipenem (IMI)	0.1 [0.0–0.4]	55	20	71%	<0.01
Meropenem (MEM)	7.9 [0.8–14.9]	41	9	85%	<0.01
***Macrolides***					
Azithromycin (AZI)	38.9 [18.4–59.3]	578	9	98%	<0.01
Erythromycin (ERY)	63.2 [33.7–92.8]	1259	8	100%	0
*Monobactams*					
Aztreonam (AZT)	37.4 [19.3–55.5]	345	9	98%	<0.01
***Penicillins***					
Amoxicillin + clavulanic acid (AMC)	34.5 [20.9–48.1]	612	16	99%	<0.01
Amoxicillin (AMX)	80.9 [71.5–90.3]	339	5	85%	<0.01
Ampicillin (AMP)	73.5 [67.1–79.8]	3031	30	98%	<0.01
Ampicillin + sulbactam (FAM)	22.6 [8.1–37.2]	169	5	96%	<0.01
Piperacillin (PIP)	47.7 [14.0–81.5]	129	4	99%	<0.01
Piperacillin + tazobactam (TZP)	8.4 [3.0–13.8]	53	8	85%	<0.01
***Phenicols***					
Chloramphenicol (CHL)	21.9 [15.0–28.8]	314	19	93%	<0.01
***Quinolones***					
Ciprofloxacin (CIP)	25.7 [18.3–33.1]	1081	38	98%	<0.01
Levofloxacin (LEV)	30.7 [17.3–44.2]	232	8	97%	<0.01
Nalidixic acid (NAL)	58.2 [47.4–69.1]	2321	24	99%	0
Norfloxacin (NOR)	31.1 [8.7–53.5]	348	7	97%	<0.01
Ofloxacin (OFX)	36.0 [1.9–70.1]	98	3	99%	<0.01
***Tetracyclines***					
Doxycycline (DOX)	40.1 [11.5–68.7]	522	4	99%	<0.01
Tetracycline (TET)	54.7 [46.9–62.6]	2272	29	96%	<0.01
***Others***					
Rifampicin (RIF)	48.8 [0.0–100.0]	124	2	100%	0
Sulfamethoxazole/trimethoprim (SXT)	50.0 [42.0–58.0]	2333	36	99%	0
Trimethoprim (TMP)	36.3 [9.6–63.0]	400	3	98%	<0.01

## Data Availability

All data relevant to this review are included in the text and references.
